# Coverage Analysis of LoRa and NB-IoT Technologies on LPWAN-Based Agricultural Vehicle Tracking Application

**DOI:** 10.3390/s23218859

**Published:** 2023-10-31

**Authors:** Hakkı Soy

**Affiliations:** Electrical and Electronics Engineering Department, Necmettin Erbakan University, 42090 Konya, Türkiye; hakkisoy@erbakan.edu.tr; Tel.: +90-332-325-2024 (ext. 4079)

**Keywords:** LoRa, NB-IoT, LPWAN, coverage, vehicle tracking, smart farming

## Abstract

This study focuses on the recently emerged Internet of Vehicles (IoV) concept to provide an integrated agricultural vehicle/machinery tracking system through two leading low power wide area network (LPWAN) technologies, namely LoRa and NB-IoT. The main aim is to investigate the theoretical coverage limits by considering the urban, suburban, and rural environments. Two vehicle tracking units (VTUs) have been designed for LoRa and NB-IoT connectivity technologies that can be used as reference hardware in coverage analysis. On this basis, the closed-form explicit analytical expressions of the maximum transmission range have been derived using the Hata path loss model. Besides, the computer simulation results have been validated via the maps from XIRIO online radio planning tool. In light of the obtained findings, several evaluations have been made to enhance the LPWAN-based agricultural vehicle tracking feasibility in smart farms.

## 1. Introduction

In recent years, we have seen the fast adoption of information and communication technologies in agricultural production globally [1,2]. This trend has significantly contributed to the spread of smart farming techniques, which allows farmers to create new opportunities to reduce their workload, improve agricultural production, and even enhance resource efficiency (e.g., seeds, water, fertilizers, and pesticides) sustainably [3,4]. The connected farm operations have enormous potential to further improve sustainability by integrating independent agricultural activities into the overall farm management systems [5]. In this context, thanks to wireless connectivity, it can be considered to significantly reduce operating costs by remotely tracking agricultural vehicles and machinery (e.g., tractors, harvesters, and threshers) for seeding and harvesting operations in large farmlands [6,7,8]. However, as the agricultural fields expand, guaranteeing reliable data delivery between vehicles and machinery becomes very difficult due to the impairments (i.e., noise, path loss, shadowing, and multipath propagation) caused by the wireless channel [9].

During the past decade, in response to the increasing demand for networked embedded system applications, the Internet of Things (IoT) continued developing as a key technology supporting the integrating of related operations and processes in several industries [10]. IoT simply means a global network infrastructure enabling near real-time monitoring of connected things. A typical IoT system comprises three basic components, i.e., end devices, a gateway, and a cloud server. The end devices collect data from sensors and send it to a gateway. A gateway is a linking bridge that forwards collected data to a cloud server. A cloud server stores and processes the overall data [11]. The widespread use of agricultural IoT allows farmers to get connected to their farms from anywhere and anytime. Therefore, the farmers can easily monitor the field conditions and crops via internet-connected computers or smartphones. Moreover, they can also control many activities remotely at different places on the farm. Consequently, agricultural activities can be automated, and operational costs can be reduced [2,4,12,13].

On the other hand, during the last decade, the emerged concept of the Internet of Vehicles (IoV) came into the spotlight as an extension of the IoT by creating opportunities to exchange data among vehicles, pedestrians, drivers, and network infrastructure [14,15]. The IoV concept has transformative potential for transportation and connected mobility ecosystem services. For instance, in smart farms, the IoV application can connect agricultural vehicles, machinery, and the farmhouse to each other. IoV-based connectivity allows gathering data from in-vehicle sensors (i.e., speed, flow, force, location, camera, etc.) and monitoring the inspections remotely. When needed, the connected agricultural vehicles can automatically send real-time data about the current operation conditions and their geographical locations. Therefore, the farmers can collaboratively organize their routine tasks with optimal resource consumption (e.g., fuel, water, fertilizer, and pesticides) [16,17].

Nowadays, a broad range of IoT connectivity technologies are available with different capabilities, data rates, and coverage to meet specific goals aimed at different applications in both environmental and industrial fields. As in many IoT-based networks, the main priority of agricultural applications is the wide coverage over the area of interest, even if the data rate remains low. Although 5G will undoubtedly bring new opportunities to smart farming in the near future, today, we have the next-generation wireless technologies that enable the IoT revolution. The Low Power Wide Area Network (LPWAN) is a promising solution for agricultural IoT applications, which require extended coverage, high scalability, and low deployment cost. LPWANs can be categorized into two main groups: 3GPP-supported technologies (e.g., EC-GSM-IoT, LTE-M, and NB-IoT) that work over the licensed spectrum and proprietary technologies (e.g., LoRa, SigFox, RPMA, Telensa) that work over an unlicensed spectrum [18,19].

LoRa and NB-IoT are prominent technologies that have recently experienced fast growth. Thanks to these popular technologies developed to deliver wireless connectivity to wide geographical areas, large-scale IoT applications can be easily implemented in smart environments (home, city, factory, grid, and farm) [20,21]. This study proposes an agricultural vehicle tracking application for smart farm management through LoRa and NB-IoT technologies by establishing an IoV system architecture. In the proposed IoV-assisted agricultural vehicle tracking application, LoRa and NB-IoT technologies have been employed to connect agricultural vehicles and machinery to a cloud server in two separate scenarios. Also, the flexible VTU hardware platform was prototyped for cloud-connected vehicle tracking among farmland borders. Considering the RF transceiver’s specifications on reference VTU hardware, the coverage range of the LoRa and NB-IoT technologies was analyzed using the Hata path loss model for different antenna heights, environmental settings (urban, suburban, and rural), and obstacle features on the test field. According to the obtained results from theoretical analysis, thanks to sub-GHz radio benefits, both LoRa and NB-IoT connectivity provide satisfactory coverage for most of the practical agricultural vehicle tracking applications. In addition, if LTE infrastructure is present in the area of need, it has been shown that NB-IoT can provide wider coverage compared to LoRa connectivity.

The remainder of the paper is organized as follows: Section 2 reviews the state-of-the-art academic studies on vehicle tracking applications based on LoRa and NB-IoT. Section 3 explains the LPWAN-based agricultural vehicle tracking application and describes using LoRa and NB-IoT technologies for wireless connectivity. Section 4 introduces the designed reference VTU hardware platforms for IoV connection. Section 5 presents the coverage analysis from different perspectives. Finally, Section 6 concludes the study and highlights the contributions presented in this research.

## 2. Related Works

This section discusses the current literature that draws on using LPWANs for geolocation tracking applications. Remarkably, most of these studies are based on LoRa technology. Down below, the recently published papers have been summarized with the aim of investigating the impact of the channel characteristics related to the coverage of wireless networks.

Baharudin and Yan presented the design of a GPS-supported location tracking system through LoRa connectivity [22]. Their designed system provides information about the GPS position (latitude and longitude), speed of mobility over the ground, and the direction of movement of mobile objects. The LoRa devices were built on the Arduino Uno platform, while the gateway runs on Intel Galileo Gen 2 board. Both were equipped with a Dragino LoRa Shield, which supports an 868 MHz frequency band. The low-cost GMS7-CR6 GPS module was also used to provide accurate satellite positioning. The data transmission reliability of the system was evaluated by considering the Received Signal Strength Indicator (RSSI). The obtained experimental results showed that the coexistence of multiple LoRa devices degrades the signal quality of received packets, especially as the number of LoRa devices moving in the same path increases.

Ramli et al. proposed an open-source tracking system that determines the location and speed of tourist boats in Malaysia [23]. The study’s main aim is to investigate the practical limits of signal transmission with LoRa technology. For this purpose, the authors designed a location tracking board (transponder) with a GPS receiver, LoRa transmitter, and Raspberry Pi. They have also used a Dragino LG01 gateway to receive the packets from boats and forward them to the server via LAN, Wi-Fi, 3G, or 4G. Although the urban and rural ranges of LoRa transmissions were 2 km and 20 km, respectively, the average limit of the gateway is at the 400-m radius, which is far below the expected range. To minimize the effects of the signal attenuation sources (e.g., buildings), the authors suggest using higher-quality antennas by improving the height and placement.

Santa et al. focused on the end-to-end network architecture that can enable vehicular monitoring and tracking applications [24]. In their proposed model, the vehicle’s current status is captured with On-Board Diagnostics (OBD)-II protocol, and the original data packets were compressed to transmit over the constrained bandwidth and transferred to a cloud server through the gateway. LoRa technology was used as the network’s backbone to establish the wireless link between the vehicles and the server. The authors also deployed an experimental test bench to demonstrate the validity of the proposed monitoring platform. Thanks to the information visualization capability of the cloud server, it is possible to track vehicles’ locations and operational parameters. The LoRa devices comprised an Arduino board and Microchip RN2483 transceiver chip. An omnidirectional antenna with a gain of 2.2 dBi was attached to each end device to transmit at 14 dBm power. On the other side, to collect the data packets from all assigned end devices, the RisingHF RHF2S008 gateway was used, which is equipped with Semtech SX1301 multi-channel concentrator chip and also 5 dBi gain omnidirectional antenna.

Murillo et al. evaluated the performance of LoRa technology in transit vehicle tracking services in medium-sized cities where they do not have exclusive lanes [25]. In this context, the authors conducted several experiments to measure the RSSI values of all the data packets a gateway receives. The experiments were repeated many times for different setups by changing the distance, the number of LoRa devices, and the main LoRa transmission parameters (spreading factor, bandwidth, and coding rate). According to the obtained results, the performance of LoRa transmission (i.e., the percentage of successfully received packets and the RSSI of received packets) was fairly variable when the distance and the number of LoRa devices were scaled. It was also shown that direct line of sight (LOS) strongly influences the performance of LoRa connectivity. When there were too many obstacles, the LoRa gateway stopped receiving packets. The best distance reached in experiments was about 890 m.

Camargo et al. experimentally explored the coverage and performance of LoRa technology for tracking a fleet of vehicles in the context of smart cities [26]. The authors stated that the success of the vehicle tracking application depends directly on its ability to send the coordinates to the Internet, even when obstacles (i.e., trees, elevated buildings, flatlands, valleys) are in their way. They also expressed that the obstacles attenuate the signal propagation, and reduce communication range. Beyond that, they also reported that the irregular terrain profile could even interrupt communication. The physical layer of LoRa technology can be up to 5 km in urban areas due to its robustness against a high degree of interference, multi-path fading, and Doppler effects. The experimental tests were realized using commercial, assembled, and programmed LoRaWAN tracker devices. A LoRa gateway with a Semtech SX1301 chip was placed on the rooftop of a building on the university campus. Both RSSI and SNR values were measured by the gateway and recorded to evaluate the influence of different vehicle speeds. The obtained results showed that the minimum radius of the gateway was 2.5 km.

Additionally, limited research studies carried out geolocation tracking with NB-IoT technology. At this point, it should be highlighted that the NB-IoT was initially used for stationary devices due to the lack of ’handover’ support mechanisms in 3GPP Release 13. In mobile networks, handover refers to a procedure of transferring an ongoing session of a mobile terminal when it moves away from one coverage to another. Due to no handover ability, LTE Cat NB1 mobile devices must re-register to the network whenever they move to a new cell. Therefore, they consume extra power, and intermittent disconnects frequently happen. Furthermore, the cell reselection procedure is initiated only after the mobile device has been completely disconnected from the last cell location. However, with the 3GPP Release 14, the enhanced NB-IoT specifications bring connection re-establishment to LTE Cat NB2 devices, allowing them to move from one BS to another without renegotiating a new connection through the ‘handover’ mechanism [27].

Santa et al. designed and implemented the LPWAN-based On-Board Unit (OBU) for personal mobility devices [28]. Unlike their previous study, the authors aimed to analyze the performance of NB-IoT technology. The designed OBU hardware was composed of two mainboards. The first board includes the CPU and memory using a small-scale computer, Raspberry Pi Zero W. The second board integrates the Lantronix A2235-H GPS receiver, the Murata CMWX1ZZABZ-093 LoRaWAN transceiver, and the Quectel BC95-G modem. The OBU was attached to an e-scooter driven between 10 and 20 km/h around the campus in the experimental study. Compared to the results of the previous work [24], the obtained results verified that the NB-IoT technology scales worsen with each base station and consume more energy but that they offer a clear advantage regarding service quality.

The main contribution of this study is introducing a theoretical approach to evaluate the transmission limits of LoRa and NB-IoT technologies for rural, suburban, and urban areas. In the considered agricultural vehicle tracking application, the LoRa and NB-IoT technologies enable transmitting GPS data at a long distance. Since the travel distance of agricultural vehicles is limited on the small- and medium-scale farmlands, the handover problem will not happen for geolocation tracking. Therefore, agricultural vehicle tracking can be realized by using the LoRa and NB-IoT technologies. In this framework, a theoretical investigation was carried out to determine the effect of antenna heights, operating frequency, fade margin, and propagation environment on transmission range. To the best of the author’s knowledge, there is no comprehensive coverage analysis of LoRa and NB-IoT connectivity technologies for agricultural vehicle tracking applications in farmlands. Therefore, the gap is planned to be addressed in this study.

## 3. LPWAN-Based Agricultural Vehicle Tracking

The agricultural vehicle tracking application helps farmers to integrate vehicles and machinery into their existing smart farm management system. This capability allows them to decide on the proper and efficient use of resources in their farming activities. Beyond that, the farmers can deploy their vehicles and machinery in an optimized way. Therefore, it can be possible to provide cost-effective routing and mobility management. The performance and success of the vehicle tracking application tightly depends on the wireless connectivity involved in the smart farm infrastructure for data transmission. When considering the tracking of vehicles and machinery on large-scale agricultural fields, the use of LPWANs comes forward thanks to their advantageous features such as large coverage, energy efficiency, and low complexity. Therefore, LoRa and NB-IoT technologies were considered to enable the proposed IoV-based agricultural vehicle tracking application.

**Figure 1:**
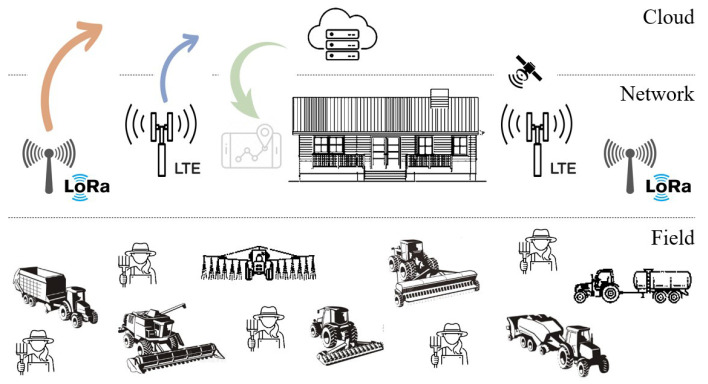
System model of the LPWAN-based agricultural vehicle tracking application.

The considered system model consists of agricultural vehicles and machinery that can be used to execute farming operations. As in a typical IoT deployment, the system architecture is separated into three layers, namely the field layer, the network layer, and the cloud layer. The field layer contains the agricultural vehicles and machinery inside and outside the farm. The network layer allows wireless connectivity through LoRa technology with proprietary (non-cellular) network implementation or NB-IoT technology with an existing cellular network of mobile operators. All collected data were aggregated by a LoRa gateway (GW) or an LTE base station (BS) and forwarded to the cloud server. Finally, the cloud layer is responsible for storing and processing the data by providing a reliable, accessible, and scalable environment. Figure 1 shows the overall model of the IoV-based agricultural vehicle tracking application.

In the considered application, vehicle tracking is enabled based on GPS services. GPS is a satellite-based navigation technology which presents precise location information of tracked assets (e.g., machinery, vehicles) just in time. GPS satellites broadcast signals that contain the information required for localization. When a GPS receiver picks up broadcasted signals from at least four satellites, it determines the geographical location on the Earth’s surface. Accordingly, the positioning data (geographic coordinate, speed, time, and velocity) can be calculated automatically [29]. Over the past decade, GPS-based applications have been largely used for precision farming activities, such as vehicle guidance, farm planning, field mapping, soil sampling, and crop scouting. GPS technology can also allow for labor tracking in large farmlands. All these applications represent a significant opportunity for farmers to be more productive and efficient in their farming businesses while simultaneously reducing environmental pollution [30,31].

On the field layer, it is assumed that each agricultural vehicle is equipped with a vehicle tracking unit (VTU), which is a printed circuit board (PCB) that carries the global positioning system (GPS) receiver and radio-frequency (RF) transceiver as well as the microcontroller and necessary components. The GPS receiver acquires signals from satellites and determines the location, speed, and direction of vehicles and machinery. RF transceiver provides a wireless connection to a LoRa GW or an LTE BS, depending on the wireless connectivity employed for agricultural vehicle tracking. Therefore, the gathered information is transmitted to the cloud server as the agricultural vehicles/machinery move. Finally, the cloud server makes data analytics on collected location data and converts the valuable information into visuals (charts, graphs, and maps) that farmers can access at any remote location with an internet connection. LoRa and NB-IoT technologies were designed to improve dense IoT networks’ coverage, energy efficiency, and scalability. However, there are essential differences between them in terms of network architecture and service of operation [32]. Accordingly, two network architectures have been introduced in conjunction with preferred LPWAN technology.

### 3.1. IoV through LoRa Connectivity

LoRa (Long Range) is an LPWAN technology that was first introduced by Cycleo in 2010 and then, in 2012, was acquired and patented by Semtech Corporation. A distinctive feature of LoRa technology is that it allows long-range data transmission for energy-restricted devices at a low data rate. LoRa-compatible devices can demodulate weak signals with their very good receiver sensitivity. LoRa was developed for sub-GHz ISM bands. Two frequency bands are available for application in the European continent that are 863–870 MHz and 433–434 MHz [33]. Conceptually, LoRa describes the PHY layer, in which the chirp spread spectrum (CSS) is used as a modulation technique. On the other hand, the MAC sub-layer, called LoRaWAN (LoRa for Wide Area Networks), enables the end devices to access GWs easily [34].

**Figure 2:**
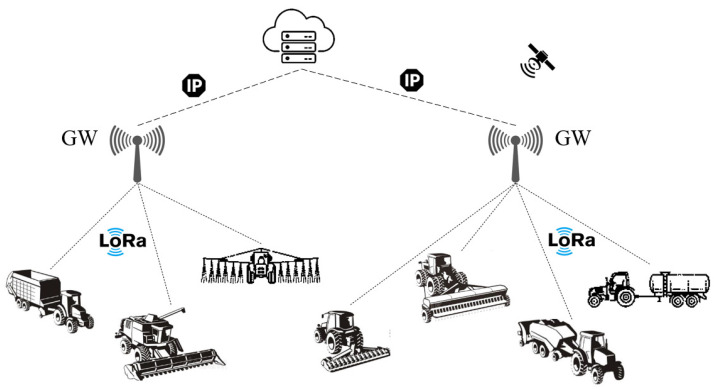
LPWAN-based agricultural vehicle tracking with LoRa connectivity.

LoRaWAN networks are deployed with star-of-stars topology in which GWs perform the bridge functions and relay the messages between the end devices and a network server. All the GWs are linked to the server through an IP connection. It is possible that multiple GWs can receive and forward the data from the same end device. But the network server filters the duplication and authenticates the end devices by selecting the most suitable GW [35]. In LoRa-based IoV network architecture, the agricultural vehicles are equipped with VTU that contains a LoRa transceiver. The GWs have an Internet connection via Ethernet or cellular network. Also, each vehicle/machinery is connected to at least one GW in the farm’s borders. Practically, large-scale farmland may be covered with a few LoRa GWs that can be accessed by agricultural vehicles. Figure 2 shows the IoV network architectures with LoRa connectivity to realize the agricultural vehicle tracking application.

### 3.2. IoV through NB-IoT Connectivity

NB-IoT is another LPWAN technology standardized by 3GPP (with Release 13, LTE Cat NB1). It is a simplified version of the LTE specification, designed for high-speed communications through widely deployed cellular infrastructure. NB-IoT uses licensed frequency bands assigned to mobile operators. There are three frequency bands available for application in the European continent: B3 (1800 MHz), B8 (900 MHz), and B20 (800 MHz). These are integrated into LTE infrastructure with a single narrow band of 200 kHz bandwidth. Due to the narrow bandwidth, the data rate peaks around 127 kbps in downlink and 159 kbps in uplink (with Release 14, LTE Cat-NB2). It should be highlighted that the NB-IoT was designed to enhance the coverage of IoT applications, especially for indoor environments and hard-to-reach geographical areas. Therefore, NB-IoT has an advantage of 20 dB coverage improvement on the link budget compared to the legacy LTE system. This means the maximum coupling loss (MCL) can be up to 164 dB [36]. On this basis, NB-IoT can allow a wider coverage of up to 40 km in lower-density rural areas with a much higher payload length of up to 1600 bytes [37].

**Figure 3:**
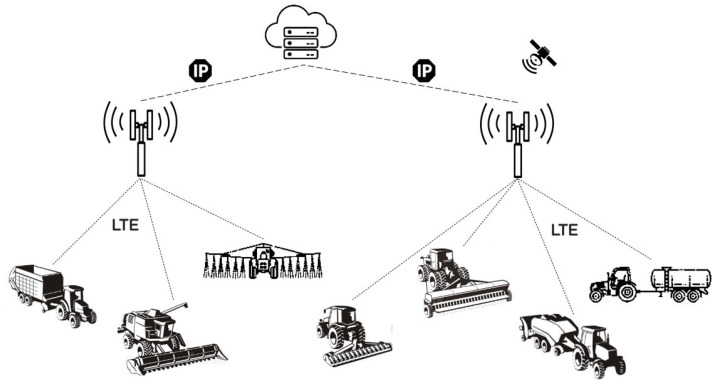
LPWAN-based agricultural vehicle tracking with NB-IoT connectivity.

The end devices are directly connected to the BS of the existing cellular network infrastructure for NB-IoT connectivity. Since this feature eliminates the dependency of an additional GWs installation, vehicle tracking can be realized with lower setup costs and higher flexibility on the farmlands. Beyond that, its robustness makes NB-IoT connectivity an ideal solution for many IoT applications [38]. In NB-IoT-based IoV network architecture, the agricultural vehicles are equipped with VTU that contains an NB-IoT transceiver. Each vehicle is connected to the nearest BS, which acts as a GW to relay the data between the agricultural vehicles and the cloud server. Compared with LoRa, NB-IoT handles two-way data traffic more simply, securely, and reliably. Also, it has the advantage of low latency. Although the burden of network subscription costs, NB-IoT offers high connection density (up to 100,000 end devices). It is noteworthy that NB-IoT connectivity depends on LTE coverage, which is only sometimes guaranteed in rural and remote suburban areas. Therefore, employment of NB-IoT technology in agricultural applications may only be valid in LTE-covered areas [18,32]. Figure 3 shows the IoV network architectures with NB-IoT connectivity to realize the agricultural vehicle tracking application.

## 4. Hardware Design

**Figure 4:**
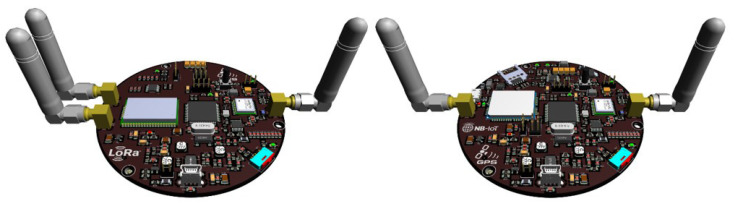
VTU prototype hardware designs for LoRa and NB-IoT technologies.

The coverage of LPWAN technologies used in the tracking application mainly depends on the wireless channel characteristics (e.g., path loss, shadowing, and multipath propagation) and the hardware capabilities of VTUs that connect agricultural vehicles to the network. Before analyzing the coverage of LoRa and NB-IoT technologies for agricultural vehicle tracking, two different hardware designs were introduced to be used as a reference for concrete comparison. As can be seen from Figure 4, both of the VTU designs have been created on the same platform, but they were equipped with different RF transceivers depending on the wireless technology used for IoV connectivity.

The designed VTU boards have been equipped with an STM32F407VGT6 microcontroller, L70-R GPS module, and additional components to provide desired functionality. A Li-Po battery of 2000 mAh 3.7 V was also used to supply a stable voltage when external power was lost. STM32F407VGT6 microcontroller is manufactured by STMicroelectronics, and it has a 32-bit ARM Cortex-M4 with an FPU core with 168 MHz frequency [39]. Quectel L70-R GPS module has a compact design, low cost, and high precision with ultra-low power consumption in tracking mode. It has 66 acquisition channels, 22 tracking channels, and assisted GPS (A-GPS) support for indoor localization. L70-R communicates with the microcontroller using the UART interface [40].

In the LoRa version of the VTU design, the Microchip RN2483 transceiver module was used to provide wireless networking capability. The RN2483 module contains a PIC18LF46K22 microcontroller with Semtech’s SX1276 transceiver. It can be operated in the 433 MHz and 868 MHz license-free frequency bands. LoRaWAN Class A protocol stack is embedded on the RN2483 module to run as an end device in the LoRa networks. The receiver sensitivity can be down to 146 dBm, and the transmitter produces output power adjustable up to +20 dBm. The manufacturer gives a 5 to 15-km transmission range in urban and suburban areas [41]. The maximum allowable transmission range varies depending on the specifications of the GW, which is used to complete the signal path from vehicles to the cloud server. Due to the complexity of both hardware design and embedded firmware development procedures, the usage of a ready-made LoRa GW is considered under the scope of this study. In coverage analysis, the Raspberry Pi single-board computer with RAK831 concentrator has been taken as reference, powered by Semtech’s SX1301 baseband processor [42].

On the other hand, the NB-IoT version of the VTU design has been equipped with a Quectel BC95-G module. BC95-G is a high-performance NB-IoT module that supports multiple frequency bands (B1:2100 MHz/B3:1800 MHz/B5:850 MHz/B8:900 MHz/B20:800 MHz/B28:700 MHz) and it can communicate with mobile network infrastructure through 3GPP Release 14. Since it has handover capability, vehicle/machine tracking can be done uninterruptedly in large-scale agricultural areas covered by more than one LTE BS. BC95-G module operates between 3.1 V to 4.2 V and communicates with the microcontroller using the UART interface. The maximum output power and receiver sensitivity values are +23 dBm and 129 dBm, respectively [43]. The signal path from vehicles to the cloud server was completed through a typical LTE BS, as described in [44].

## 5. Coverage Analysis

In this section, depending on the designed VTU hardware platforms, the coverage analysis was carried out to reveal the approximate transmission limits of LoRa and NB-IoT technologies. For a typical wireless network, the coverage refers to the maximum distance that enables the received signals to exist above a certain threshold level. For a receiver to detect the desired signal, the RSSI must be greater than the receiver sensitivity [45]. The receiver sensitivity is the minimum signal power level to correctly receive the packets with an acceptable packet error rate (PER). In general, it is expected that the PER should be less than 1% [46]. The higher receiver sensitivity means better coverage to receive signals from further away [47].

The link budget summarizes a communication link that allows for calculating the coverage range of a wireless system. It includes all the gains and losses throughout the propagation path from the transmitter to the receiver [48]. By using the link budget, the received signal power can be calculated as follows:

(1) $$P_{r}=P_{t}-L_{t}+G_{t}-PL+G_{r}-L_{r}$$

where $P_{t}$ is the transmitter output power (dBm), PL is the path loss (dB), $G_{t}$ and $G_{r}$ are the antenna gains (dBi), and $L_{t}$ and $L_{r}$ are the losses (dB) from cables/connectors at transmitter and receiver, respectively. Accurately calculating the link budget is critical to provide reliable data transmission. To estimate the coverage range, the maximum allowable path loss can be calculated from the link budget equation as follows:

(2) $$PL_{max}=P_{t}-L_{t}+G_{t}-S+G_{r}-L_{r}-m$$

where *S* is the receiver sensitivity (dBm) and *m* is the fade margin (dB) due to the obstructions and atmospheric attenuation. Clearly, the fade margin is the difference between the RSSI and receiver sensitivity. It should be high enough to guarantee the RSSI is over the receiver sensitivity. Depending on the propagation environment, a certain margin should be reserved to make the link budget more reliable. When considering the unpredictable signal fading on the wireless channel, the fade margin is usually taken in the range of 10–30 dB [46].

**Figure 5:**
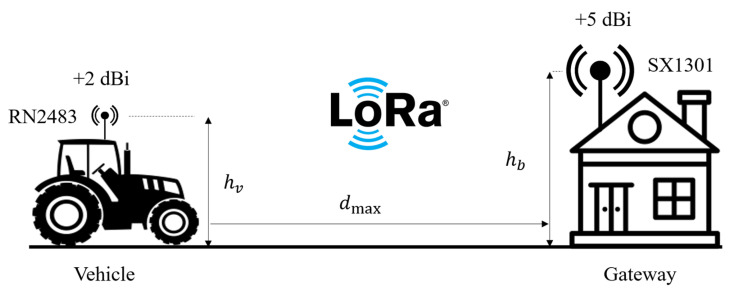
LoRa uplink channel from vehicle to GW.

**Figure 6:**
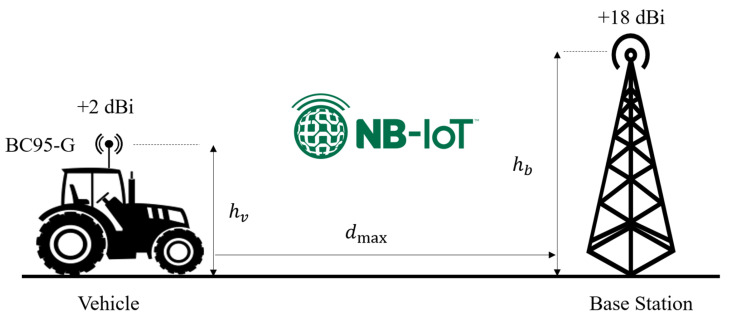
NB-IoT uplink channel from vehicle to BS.

In the considered agricultural vehicle tracking application, the uplink channels for LoRa and NB-IoT connectivity technologies have been visualized in Figure 5 and Figure 6, respectively. According to that, the link budgets can be calculated using LoRa and NB-IoT transceiver modules’ specifications on their datasheets [41,43]. Table 1 summarizes the utilized operating parameters of the VTU designs at the transmitting side. Besides, Table 2 summarizes the uplink budget for LoRa and NB-IoT connectivity by using a GW (with SX1301) and LTE BS operating parameters (i.e., receiver sensitivity, connector losses, and antenna gains) on the receiving side. It is especially noteworthy that, for LoRa connectivity, the highest spreading factor (SF12) was used to maximize the transmission range due to its high link budget capability [49].

Path loss is a major characteristic of wireless channels that represents the attenuation of the radiated signal from a transmitter to a receiver due to the traveled distance. Therefore, the key factor for the link budget calculation is the path loss model [50]. Depending on the nature of the propagation environment, several path loss models have been introduced in the literature [51]. The Hata model (also known as the Okumura-Hata model) is widely used to predict propagation path loss in urban areas. The Hata model also has an extended formulation for path loss in suburban and rural areas. It should be emphasized that the Hata model is applicable in the range of frequency from 150 MHz to 1500 MHz, and it is suitable for a coverage range of up to 100 km [52]. In urban areas, the Hata model gives the path loss as follows [53].

(3) $$\begin{array}{cc} PL_{Urban} & =69.55+26.16\times \log_{10}f-13.82\times \log_{10}h_{b}-\phi \\ \; & +\left(44.9-6.55\times \log_{10}h_{v}\right)\times \log_{10}d \end{array}$$

where *d* is the distance, ϕ is the correction factor, $h_{b}$ is the GW/BS antenna height, $h_{v}$ is vehicle (or machinery) antenna height, and *f* is the frequency in MHz. The correction factor was given as follows:

(4) $$\phi =3.2\times {\left[\log_{10}h_{v}\right]}^{2}-4.92.$$

The Hata model also specifies the path loss for suburban and rural areas as follows [53]:

(5) $$PL_{Suburban}=PL_{Urban}-2\times {\left(\log_{10}\left(\frac{f}{28}\right)\right)}^{2}-5.4,$$

(6) $$PL_{Rural}=PL_{Urban}-4.78\times {\left(\log_{10}f\right)}^{2}+18.33\times \log_{10}f-40.98.$$

It should be emphasized that the correction factor is different for rural/suburban areas, and it can be calculated as follows:

(7) $$\phi =0.8+\left(1.1\times \log_{10}f-0.7\right)\times h_{v}-1.56\times \log_{10}f.$$

According to path loss expressions as a function of distance, the coverage of LoRa and NB-IoT links can be calculated from the $10^{\psi }$ formula. Therefore, considering the different propagation environments, ψ parameter can be derived as follows:

(8) $$\begin{array}{cc} \psi_{Urban} & =\frac{PL_{Urban}-74.47-26.16\times \log_{10}f-13.82\times \log_{10}h_{b}}{44.9-6.55\times \log_{10}h_{v}}\\ \; & +\frac{3.2\times {\left[\log_{10}h_{v}\right]}^{2}}{44.9-6.55\times \log_{10}h_{v}}, \end{array}$$

(9) $$\begin{array}{cc} \psi_{Suburban} & =\frac{PL_{Suburban}-63.35-27.72\times \log_{10}f-13.82\times \log_{10}h_{b}}{44.9-6.55\times \log_{10}h_{v}}\\ \; & +\frac{\left(1.1\times \log_{10}f-0.7\right)\times h_{v}+2\times {\left(\log_{10}\left(\frac{f}{28}\right)\right)}^{2}}{44.9-6.55\times \log_{10}h_{v}}, \end{array}$$

(10) $$\begin{array}{cc} \psi_{Rural} & =\frac{PL_{Rural}-27.77-46.05\times \log_{10}f-13.82\times \log_{10}h_{b}}{44.9-6.55\times \log_{10}h_{v}}\\ \; & +\frac{\left(1.1\times \log_{10}f-0.7\right)\times h_{v}+4.78\times {\left(\log_{10}f\right)}^{2}}{44.9-6.55\times \log_{10}h_{v}}. \end{array}$$

The obtained equations show that the transmission range can be determined depending on the path loss, antenna heights, and frequency. When these parameters are known, the maximum transmission distance can be estimated by using the link budget calculation on the uplink channel.

**Figure 7:**
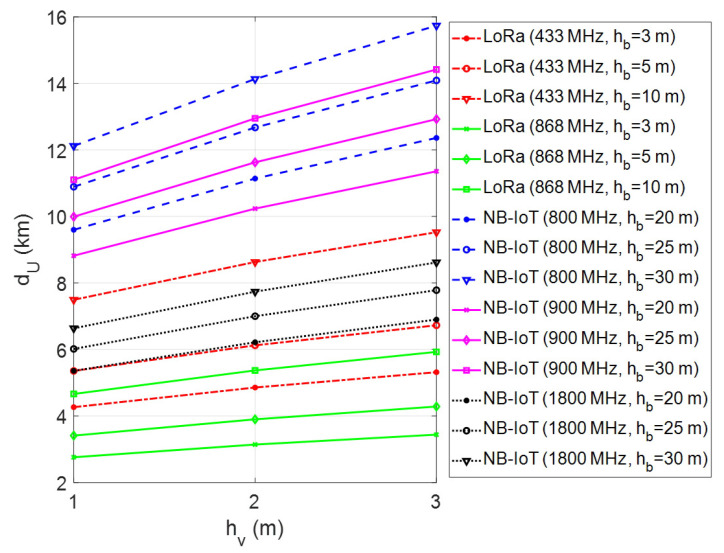
Coverage analysis of LoRa and NB-IoT technologies in urban areas.

**Figure 8:**
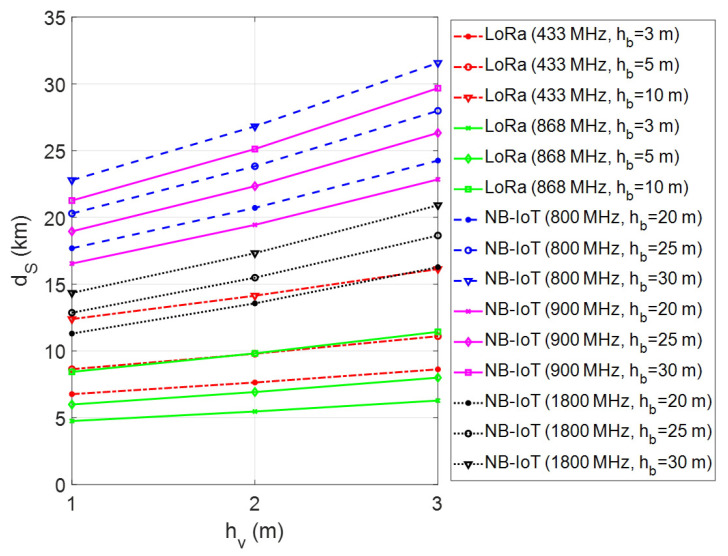
Coverage analysis of LoRa and NB-IoT technologies in suburban areas.

**Figure 9:**
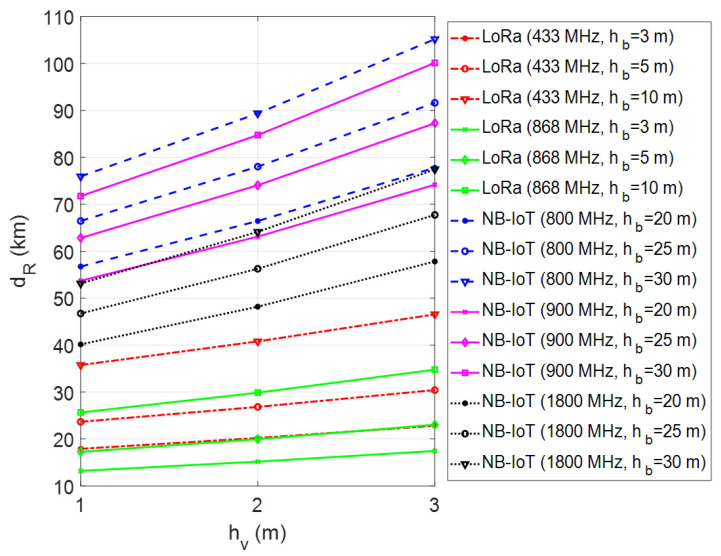
Coverage analysis of LoRa and NB-IoT technologies in rural areas.

In the presented framework, the coverage range of a single GW for LoRa connectivity and a single LTE BS for NB-IoT connectivity has been investigated through computer simulations using MATLAB software. In performed simulations, firstly the maximum transmission range has been calculated by changing the vehicle/machinery and GW/BS antenna heights for different environmental conditions. The simulations have been repeated for each frequency band of LoRa and NB-IoT deployments in Europe. The obtained results have been given in Figure 7, Figure 8 and Figure 9 for urban, suburban, and rural areas, respectively. As the plots show, the transmission range increases with antenna height for vehicle/machinery and GW/BS. The simulation results clearly show that the NB-IoT has better coverage when compared to LoRa for all urban, suburban, and rural environments. In the 900 MHz band, the NB-IoT transmission range varies from 9 km to over 14 km in urban and 50–100 km in rural environments. On the other side, in the 868 MHz frequency band, the LoRa’s transmission range may be expected to be approximately 6 km in dense urban sites and up to 35 km in rural areas. It is clear that the LoRa coverage increases at the 433 MHz band due to the better propagation advantage. According to that, the transmission range varies from 4 km to over 10 km in urban sites and up to 45 km in rural environments in the 433 MHz frequency band. On the contrary, in the 1800 MHz band, the NB-IoT transmission range is relatively short compared to sub-GHz frequencies. Note that all simulations have been made without considering the fade margin parameter in the case of m=0 dB, which introduces the theoretical upper limits of the coverage range.

**Figure 10:**
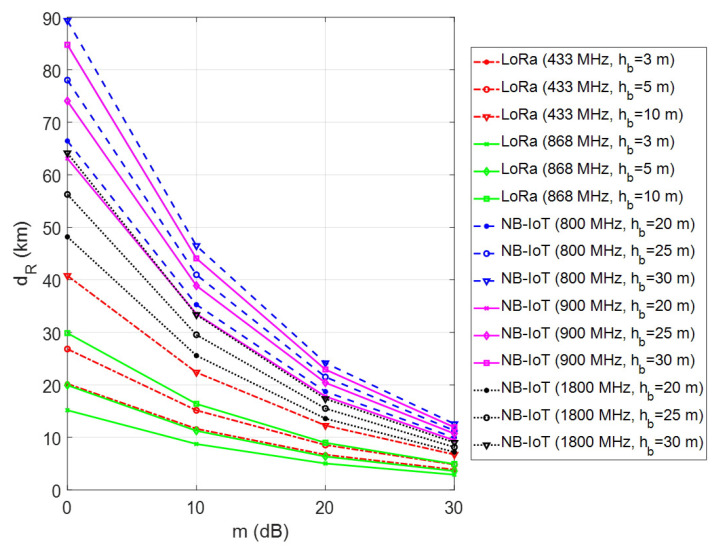
Coverage analysis of LoRa and NB-IoT technologies for different fade margins.

It should be highlighted that the path loss mainly depends on the terrain contour and topography, and the height of antennas in realistic propagation scenarios. Depending on the nature of the propagation environment, the multipath propagation and shadowing effects may simultaneously occur on the wireless channel. Especially the disruptive impact of shadowing causes signal attenuation, and thereby the wireless network coverage is significantly affected due to environmental obstacles. To investigate the effect of fade margin on the transmission range, computer simulations were repeated by changing the fade margin parameter from 0 to 30 dB for a fixed vehicle antenna height, $h_{v}=2$ m. In this simulation setup, the environment was assumed as rural, and the antenna heights of LoRa GW and LTE BS were also changed from 3 m to 10 m and 20 m to 30 m, respectively. As can be seen from Figure 10, both LoRa and NB-IoT coverage ranges significantly decrease due to objects obstructing the propagation path between the transmitter and receiver. When considering the harsh channel conditions for m=30 dB, the LoRa transmission range is under 10 km for the 433 MHz bands. However, NB-IoT still achieves a better transmission range of up to 15 km for 800 MHz bands. It is noteworthy that when the fade margin increases, the importance of GW/BS antenna height becomes less effective, and the coverage enhancement remains negligible.

**Figure 11:**
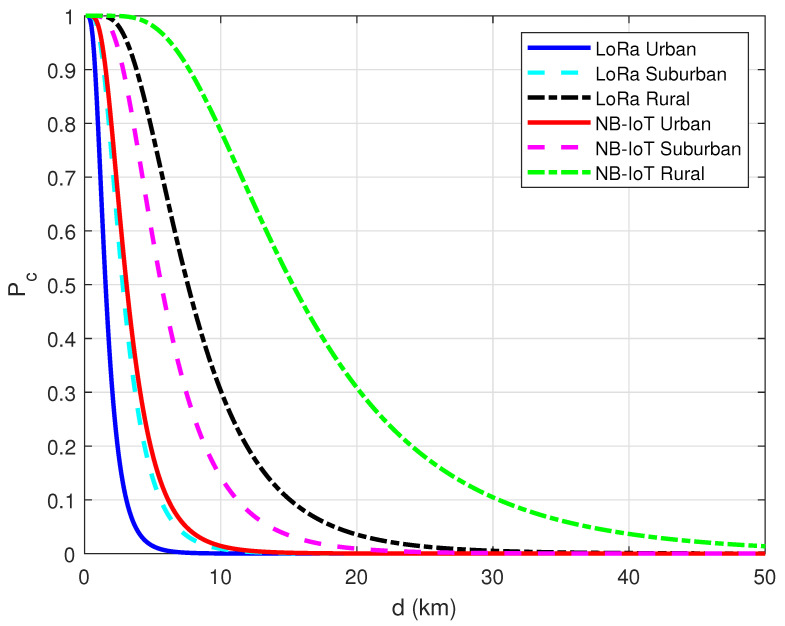
Connection probability for LoRa and NB-IoT under Hata path loss model.

To complement the theoretical coverage analysis, the connection probability between the agricultural vehicle/machinery and GW/BS was also investigated based on the Hata path loss model in urban, suburban, and rural radio propagation environments. Connection probability measures the randomness and uncertainty of achieving successful packet transmission [54,55,56]. When considering the multiple vehicles located at different positions on a large-scale farm, the connection probability of an individual one can be calculated depending on its distance to a GW/BS as follows:

(11) $$P_{c}\left(d\right)=\mathrm{Pr}\left\{PL\left(d\right)<\;PL_{max}\right\},$$

where $PL_{max}$ is the maximum allowable path loss in Equation (2). Therefore, the end-to-end connection probability can be measured using the error function definition as follows:

(12) $$P_{c}\left(d\right)=\frac{1}{2}\left[1-\mathrm{erf}\left(\frac{PL\left(d\right)-PL_{max}}{\sqrt{2}\sigma }\right)\right],$$

where $\mathrm{erf}\left(t\right)=\frac{2}{\sqrt{\pi }}\int_{0}^{t}e^{-x^{2}}dx$ and σ is the standard deviation of the Gaussian distribution that describes the shadowing effect in dB. Based on the presented formulation, computer simulations were conducted for both the LoRa and NB-IoT connectivity to reveal the effect of the propagation environment on connection probability. In this simulation setup, the operating frequency was selected as 868 MHz for LoRa and 900 MHz for NB-IoT, respectively. In addition, the antenna heights were taken as $h_{v}=2$ m, $h_{b}=5$ m for LoRa and $h_{v}=2$ m, $h_{b}=20$ m for NB-IoT, respectively. Lastly, σ was set to 10 dB to identify the shadow-fading. The results acquired in the simulation study are shown in Figure 11. As seen from the plots, NB-IoT connectivity achieves better connection probability than LoRa for all environments. However, it was observed that shadowing-induced irregularities in the wireless channel significantly affect the covered area due to the presence of randomness in the radio propagation environment.

Another simulation study was also performed to show the effectiveness of the theoretical analysis. In this way, the obtained simulation results were validated through the use of the XIRIO Online radio planning tool from Aptica [57]. XIRIO Online has worldwide high-resolution urban/rural mapping data that allows us to approximately predict the coverage of a typical LoRa GW or LTE BS. It has been empowered with a calculation engine, which is updated with the most widely used and internationally recognized propagation models (Rec. ITU-R P.526, Deygout, LOS, Rec. ITU-R P.1546, Rec. ITU-R P.530, Okumura-Hata, XIA-Bertoni, etc.). The simulation location was set to Konya Province, Turkey’s agricultural capital city. Konya has large farmland areas, most of which can be easily and effectively used for agriculture due to the ground’s fertility. It was assumed that the GW/BS is positioned in the rural area of Çumra District, which is located about 40 km away from the Konya city center. The coverage analysis was conducted considering the 868 MHz and 900 MHz frequency bands for LoRa and NB-IoT, respectively. The antenna gain and height settings are given in Table 3.

**Figure 12:**
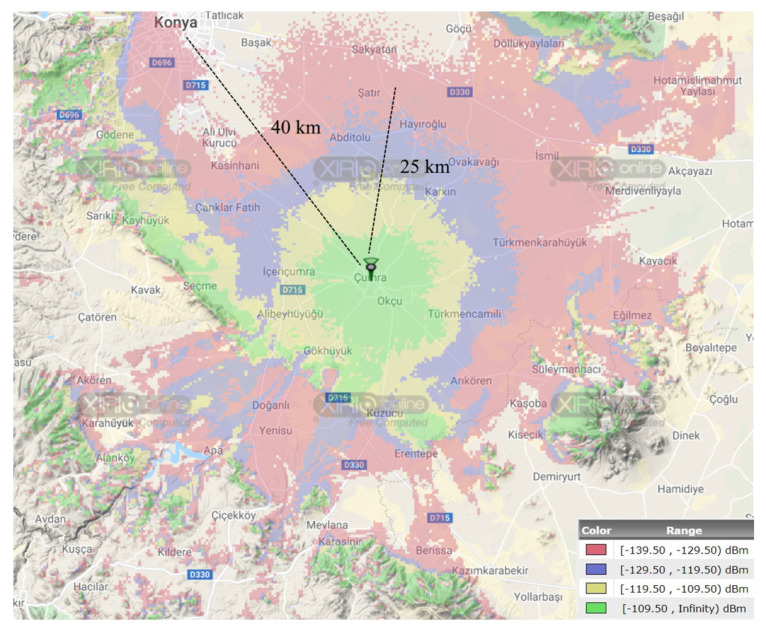
The coverage analysis of LoRa connectivity with XIRIO online tool.

**Figure 13:**
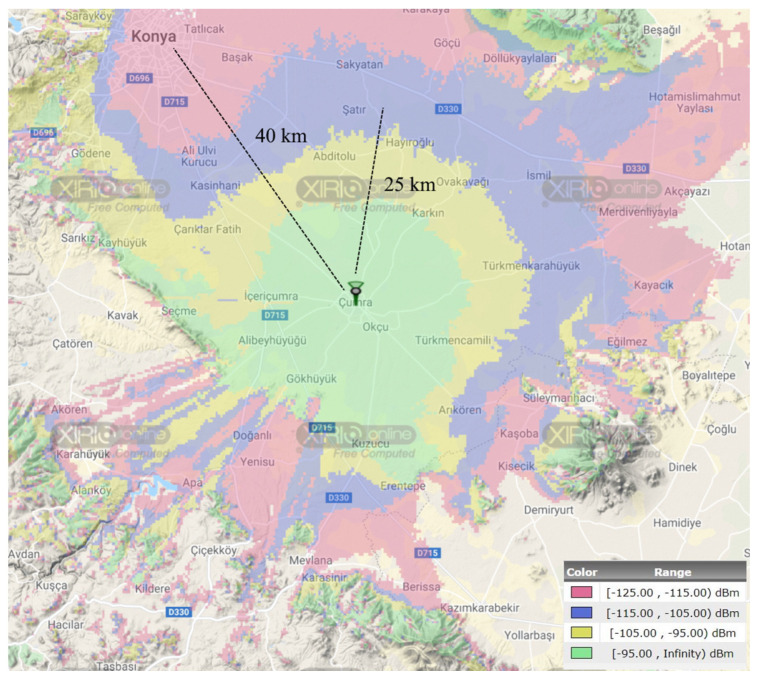
The coverage analysis of NB-IoT connectivity with XIRIO online tool.

Figure 12 and Figure 13 show the coverage maps for two different scenarios through LoRa and NB-IoT connectivity technologies, respectively. A coverage map shows the geographic area where a receiver will obtain a good reception when the received signal strength exceeds the sensitivity threshold. In the obtained maps, the covered areas were separated into four color groups (green, yellow, blue, and red) as the power level increased by 10 dBm. Apart from these, the locations outside the coverage area where the received signal is below the receiver sensitivity level were left uncolored. In the green-colored regions, it is possible to take a strong signal due to providing a better link budget availability. Towards the red-colored regions, the received signal strength decreases gradually. To easily compare the coverage maps, two reference distances (25 km and 40 km) are marked on the coverage maps. It is obvious that the NB-IoT connectivity has an extended coverage area compared with LoRa connectivity. Moreover, it is possible to provide coverage at longer distances (more than 50 km) for both LoRa and NB-IoT technologies when rough terrain features (e.g., hills, forests, mountains) permit receiving signals via unobstructed line-of-sight (LOS) paths.

## 6. Conclusions

This study provides the coverage range analysis of the LoRa and NB-IoT technologies for LPWAN-based agricultural vehicle tracking applications. The main contribution is comparing the upper bounds of LoRa and NB-IoT technologies in urban, suburban, and rural propagation environments by deriving explicit mathematical expressions. On this basis, the comparative analysis was made using the Hata path loss model, which investigates the power decay based on the frequency and antenna heights. Also, to show the effect of terrain irregularities on the coverage range, the obtained simulation results were supported by the coverage maps from the XIRIO Online radio planning tool. From the presented theoretical coverage analysis, it can be concluded that the NB-IoT provides extended coverage compared to the LoRa. Since agricultural activities are usually carried out in rural areas worldwide, both the LoRa and NB-IoT technologies are viable solutions for vehicle-tracking applications in large agricultural fields. Thanks to its cellular network independent cloud access opportunity, LoRa connectivity offers a more cost-effective operation without paying monthly access fees to mobile operators for connected vehicles. Besides, NB-IoT connectivity can be attractive due to the service continuity guarantee advantage via existing mobile communication infrastructure when LTE BS is deployed in the agricultural vehicles’ coverage area.

## Figures and Tables

**Table 1:** RF transceiver specifications of designed VTUs.

	LoRa	NB-IoT
RF Transceiver	Microchip RN2483	Quectel BC95-G
Frequency	433, 868 MHz	800, 900, 1800 MHz
Bandwidth	125 kHz	180 kHz
Transmit power	+14 dBm	+23 dBm
Receiver sensitivity	−146 dBm	−129 dBm

**Table 2:** Link budget for uplink channel of LoRa and NB-IoT connectivity technologies.

		LoRa	NB-IoT
	$P_{t}$ (dBm)	+14	+23
Vehicle	$L_{t}$ (dB)	0.5	0.5
	$G_{t}$ (dBi)	2	2
Channel	*m* (dB)	0 …30	0 …30
	$G_{r}$ (dBi)	5	18
GW/BS	$L_{r}$ (dB)	0.5	3
	*S* (dBm)	−139.5 (SF12)	−125
	$PL_{max}$ (dB)	159.5 …129.5	164.5 …134.5

**Table 3:** The antenna parameters used in the XIRIO online tool.

	Antenna Height (m)	Antenna Gain (dBi)
Vehicle/Machinery	2	2
LoRa GW	5	5
LTE BS	25	18

## Data Availability

Not applicable.

## Abbreviations

The following abbreviations are used in this manuscript:

BS	Base Station
dB	Decibel
dBi	Decibel-isotropic
dBm	Decibel-milliwatts
GPS	Global Positioning System
GW	Gateway
IoT	Internet of Things
IoV	Internet of Vehicles
LoRa	Long Range
LoRaWAN	LoRa Wide-Area Networks
LPWAN	Low Power Wide Area Network
LTE	Long-Term Evolution
MHz	Megahertz
NB-IoT	Narrow Band Internet of Things
PER	Packet Error Rate
RPMA	Random Phase Multi Access
VTU	Vehicle Tracking Unit
